# Should internal mammary lymph node sentinel biopsy be performed in breast cancer: a systematic review and meta-analysis

**DOI:** 10.1186/s12957-019-1683-8

**Published:** 2019-08-05

**Authors:** Jing Gong, Yongfu Yu, Gaosong Wu, Congyao Lin, Xin Tu

**Affiliations:** 1 Department of Thyroid and Breast Surgery, Zhongnan Hospital of Wuhan University, 169 DongHu Road, Wuhan, 430071 People’s Republic of China; 2 Department of Clinical Epidemiology, Aarhus University, Olof Palmes Allé 43-45, 8200 Aarhus N, Denmark; 3 School of Mechanical Engineering and Electronic Information, China University of Geosciences, 388 Lumo Road, Wuhan, 430074 People’s Republic of China

**Keywords:** Internal mammary lymph node, Sentinel lymph node biopsy, Breast cancer, Meta-analysis, Systematic review, Breast Neoplasms, Lymphatic metastasis

## Abstract

**Purpose:**

Results from studies of internal mammary lymph node sentinel biopsy are inconsistent.

**Methods:**

A comprehensive literature search was conducted in MEDLINE, EMBASE, Scopus, Cochrane database, and Clinical Trials. Studies reporting the rate of internal mammary lymph node sentinel biopsy (IMN-SLNB) positivity were identified. We performed pooled proportion meta-analysis using random-effects meta-analyses. The correlation of IMN and axillary lymph node (AXN) metastasis was also investigated.

**Results:**

After application of inclusion and exclusion criteria, a total of 18 articles (total number of patients = 2427) were included. The pooled estimate for IMN-SLNB positivity rate was 15% (95% confidence interval (CI) 12–17%). Significant between-study heterogeneity was observed. Our results indicate that axillary lymph node metastasis is a strong predictor of IMN involvement (OR 6.01, 95% CI, 3.49, 10.34).

**Conclusion:**

Internal mammary lymph nodes metastasis might be underestimated. Patients with positive axillary lymph nodes have a higher risk of internal lymph nodes metastasis. As a result, IMN-SLNB might be considered in these patients. Future work needs to be done to assess whether pathological confirmed IMN metastasis can affect patients’ survival.

## Background

Internal mammary lymph node (IMN) is an important part of lymphatic drainage of breast cancer. In the last few decades, a lot of effort has been made to optimize the management of IMN. It was well established in the 1970s that extended radical mastectomy (ERM) including routine dissection of IMN would not improve disease control whiles bring about serious complications [1]. Multiple studies have shown that patients with IMN metastasis had a worse outcome [2–4]. The metastasis of IMN has been incorporated into the American Joint Committee on Cancer (AJCC) cancer staging system of breast cancer since the 6th edition as an evidence of increased tumor burden.

The MA.20 [5] and the EORTC 22922–10925 trial [6] and a French trial [7] have found out that radiation therapy (RT) to IMN basin will improve patients’ survival. The National Comprehensive Cancer Network (NCCN) Breast Cancer Clinical Practice Guideline has raised the level of evidence for the recommendation of RT when there is a high risk of IMN involvement. However, it was not reliable to identify patients with IMN metastasis by clinical factors such as tumor location or axillary lymph nodes (AXN) status. As a result, patients with actual IMN involvement might escape RT, while patients without IMN metastasis would undergo unnecessary RT.

Recent studies have demonstrated that IMN status can be safely determined through sentinel lymph node biopsy (SLNB) technique [8, 9]. But questions remain whether IMN-SLNB should be performed routinely and which patient would benefit the most. To date, there have been a growing number of studies trying to answer these questions. However, many of these researches have been retrospective in nature and not much large randomized trials were performed. A previous systematic review in 2012 included 18 studies reporting that positive IMN are reliable predictors of axillary lymph node involvement. Nonetheless, this study suffered from a paucity of data and small case series and case reports have to be included [10]. In 2013, another meta-analysis enrolled 15 articles did not specify SLNB as an inclusion criterion while evaluating IMN metastasis-associated outcomes [11]. Since the publication of those two meta-analyses, more studies have become available, which justifies a new systematic review.

Our objective is to conduct a quantitative analysis of current evidence and provide important insights into the IMN management.

## Material and methods

### Study identification

We performed a computer-based literature search of PubMed/MEDLINE, Clinical trials, EMBASE, Scopus, Cochrane electronic databases, and Google Scholar. The core search strategy was as follows: internal mammary [All Fields] AND (“lymph nodes” [MeSH Terms] OR “lymph” [All Fields] AND “nodes” [All Fields]) OR “lymph nodes” [All Fields]). The following search terms were also used: extra-axillary lymph nodes, intramammary lymph node, intraMSLN, and retromammary lymph nodes. The search was neither limited by language nor by date. The full texts of all the relevant studies were retrieved. A manual search was also conducted to scan the reference list of relevant articles in order to identify additional articles. The last search was made in April 2019.

### Eligibility criteria

Studies were included in our systematic review if they fulfilled the following criteria: published research articles including patients underwent IMN-SLNB and reported the status of IMN as well as axillary lymph nodes (AXN). We excluded case reports, reviews, comments, letter to editors, correspondence, conference posters. We also excluded studies that included patients receiving neoadjuvant therapy, and studies reported extra-axillary lymph nodes other than IMN, and studies that did not report enough adequate data or pathological results.

### Data extractions and management

Data was extracted independently by two authors on a predefined form. In addition to IMN and AXN status, information on potentially confounding study-level characteristics (stage, age, length of follow-up, SLNB technique, tumor histology, tumor size, and tumor location) was also extracted with the intention of performing possible subgroup analyses. Differences between reviewers were resolved by discussion. When certain events were not explicitly stated in the study, information was calculated through available data. Several authors were contacted for unpublished data through emails when there was not enough data amenable.

### Data synthesis and meta-analysis

We calculated the pooled proportion of positivity of IMN-SLNB with the corresponding 95% confidence intervals (CI). The statistical heterogeneity across studies was tested by *Q* statistics. An alpha value of 0.5 was taken to indicate between-study heterogeneity, which is represented by *I*^2^ values. Significant between-group heterogeneity was observed and random-effects models were applied. Pooled estimated proportion of IMN-SLNB was presented by forest plots. We also calculate the odds ratio (OR) of 4 subgroups (IMN+/AXN+, IMN+/AXN−, IMN−/AXN−, IMN−/AXN−). The OR was further combined in order to explore the association of IMN and AXN. Statistical analyses were conducted in STATA 14 software (StataCorp LP, College Station, TX).

## Results

### Literature search and study identification

**Fig. 1:**
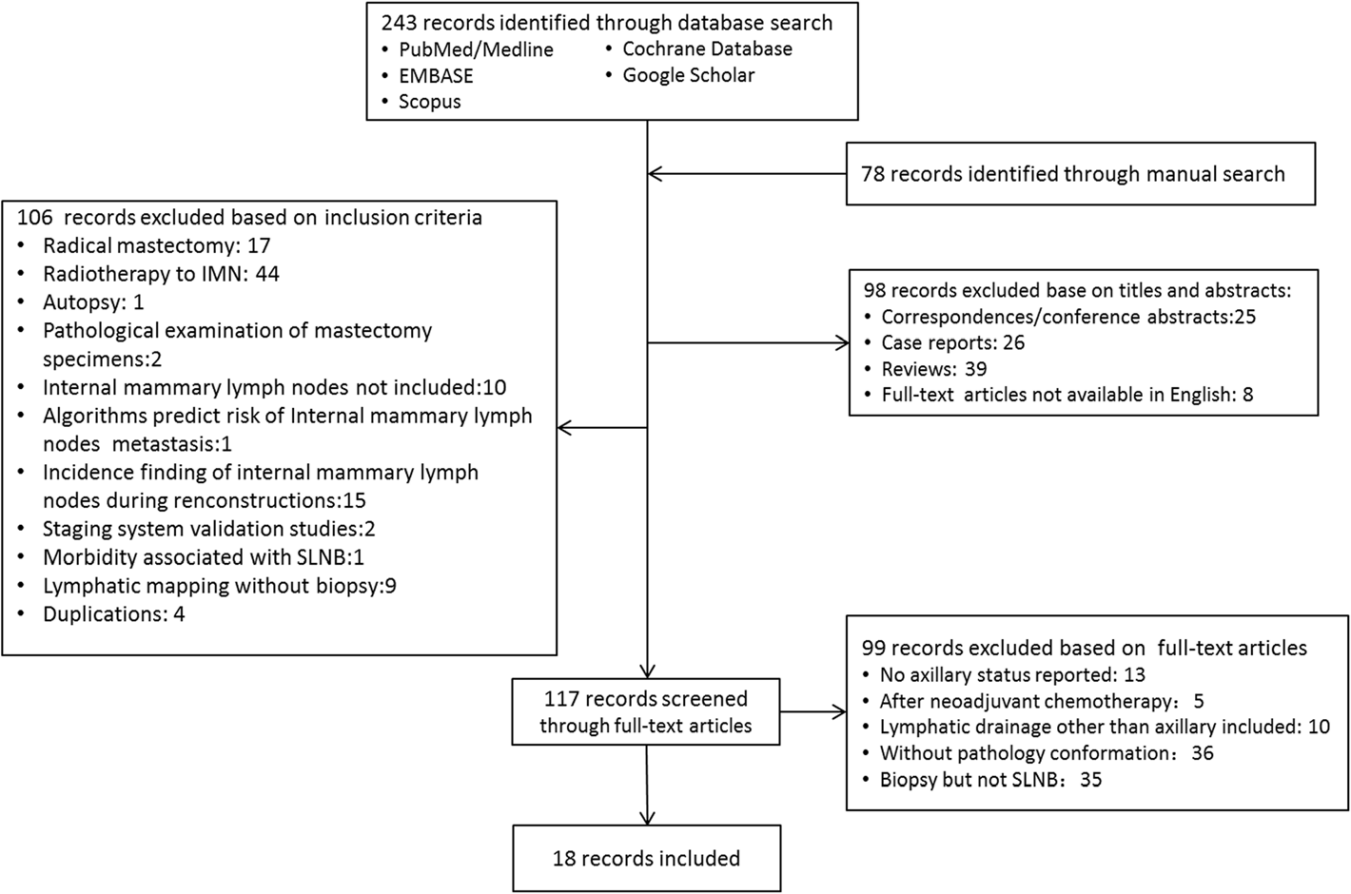
Flowchart of included studies

A systematic literature search was performed to identify studies that met the predefined eligibility criteria. The search yielded 215 articles. Full texts of 99 articles were retrieved. We defined studies reporting no more than 4 cases as small case series. Small case series and reviews, correspondence and conference posts were excluded. Studies based on the same population or on human cadavers were excluded. Furthermore, studies that enrolled neoadjuvant treatment or did not report enough data were excluded. Finally, 18 studies (with 2427 patients) were included in this systematic review. The process of research screening and selection is shown in a flow chart (Fig. 1).

### Study characteristics

Table 1 showed an overview of the characteristics of all selected articles. The majority of them was retrospective cohort studies and employed more than one method of SLNB. It should be noted that not all included studies reported the visualization rate and success rate of IMN-SLNB. Interestingly, both the visualization and success rate seemed to vary widely from study to study. IMN was visualized in 20% of patients (range 5.4–87.9%). IMN-SLNB was successful in 90.3% patients (range 45–97.3%). In spite of this, the positivity rate of IMN-SLNB was rather stable (range 8.8–25%). The highest metastatic rate was reported by Avisar et al. with 44.1% of the lesions located in the central area [12]. The lowest positive rate was reported by an early innovative study by Galimberti et al. when IMN were sampled without the help of a gamma probe [16].

**Table 1:** Characteristic of eligible studies in the meta-analysis

Author	Year	Country	No. of patients	Nature	Stage	Lymphatic mapping technique	Positivity rate (%)	IMN+		IMN-	
								AXN+ *n* (%)	AXN− *n* (%)	AXN+ *n* (%)	AXN− *n* (%)
Avisar [12]	2008	American	31	Prospective	T1-3 N0	Gamma probe or blue dye or LS	25	5 (17.86)	2 (7.14)	5 (17.86)	16 (27.59)
Bourre [13]	2009	France	161	Petrospective	T1 N0	Gamma probe and blue dye and LS	11	11 (6.83)	7 (4.34)	7 (4.34)	135 (86.25)
Carcoforo [14]	2006	Italy	65	Retrospective	N0	Gamma probe and blue dye and LS	15.40	3 (10.34)	1 (3.45)	4 (13.79)	21 (72.41)
Caudle [8]	2014	American	71	Retrospective	N0	Gamma probe and blue dye and LS	15	4 (5.63)	7 (9.86)	12 (16.90)	48 (67.61)
Cong [15]	2016	China	145	Prospective	I-IV	Gamma probe and LS and fluorescence	12.40	11 (7.01)	8 (5.10)	59 (37.58)	79 (50.32)
Galimberti [16]	2002	Italy	160	Retrospective	I-IV	LS	8.80	10 (6.25)	4 (2.50)	45 (28.13)	101 (63.13)
Generich [9]	2014	American	122	Retrospective	Tis-T2 N0	Gamma probe and LS	10	10 (9.26)	2 (1.85)	20 (18.52)	76 (70.37)
He [17]	2010	China	94	Retrospective	T1-2 N0	Gamma probe and LS and carbon nanoparticles	24	19 (20.21)	4 (4.26)	10 (10.64)	61 (64.89)
Heuts [18]	2009	Netherlands	139	Retrospective	cN0	Gamma probe and blue dye and LS	22	22 (15.83)	9 (6.47)	43 (30.94)	65 (46.76)
Lee [19]	2013	Korea	31	Retrospective	cN0	NR	20.00	1 (10.00)	1 (10.00)	0 (0.00)	8 (80.00)
Leidenius [20]	2006	Finland	122	Retrospective	T1-2 N0	Gamma probe and LS	15	10 (7.25)	8 (5.80)	28 (20.29)	92 (66.67)
Mansel [21]	2004	UK	31	RCT	cN0	Gamma probe and LS	13	2 (6.45)	2 (6.45)	15 (48.39)	12 (38.71)
Maraz [22]	2014	Hungary	77	Retrospective	T1-3 N0	Blue dye and LS	18	2 (2.60)	12 (15.58)	14 (18.18)	49 (63.64)
Ozmen [23]	2015	Turkey	72	Retrospective	cN0	LS	14	9 (12.50)	1 (1.39)	24 (33.33)	38 (52.78)
Piato [24]	2016	Brazil	20	Prospective	I-II	LS and PET-CT	21	2 (10.53)	2 (10.53)	1 (5.26)	14 (73.68)
Postma [25]	2012	Netherlands	86	Retrospective	T1-2 N0	Gamma probe and blue dye and LS	16.00	7 (8.14)	7 (8.14)	25 (29.07)	47 (54.65)
Qi [26]	2018	China	337	Retrospective	M0	NR	18.70	62 (18.40)	1 (0.30)	132 (39.17)	142 (42.14)
Veronesi [27]	2008	Italy	663	Retrospective	I-IV	Gamma probe and LS	10.30	51 (7.69)	17 (2.56)	182 (27.45)	413 (62.29)

*AXN* axillary lymph nodes, *IMN* internal mammary lymph node, *LS* lymphoscintigraphy, *M0* no presence of distant metastasis, *N0* no presence of lymphatic involvement, *NR* not reported, *PET-CT* positron emission tomography–computed tomography, *RCT* randomized clinical trial, *Tis* carcinoma in situ, *n* number of patients, *+* positive, presence of lymph node metastasis, *−* negative, no presence of lymph node metastasis

#### Meta-analysis

**Fig. 2:**
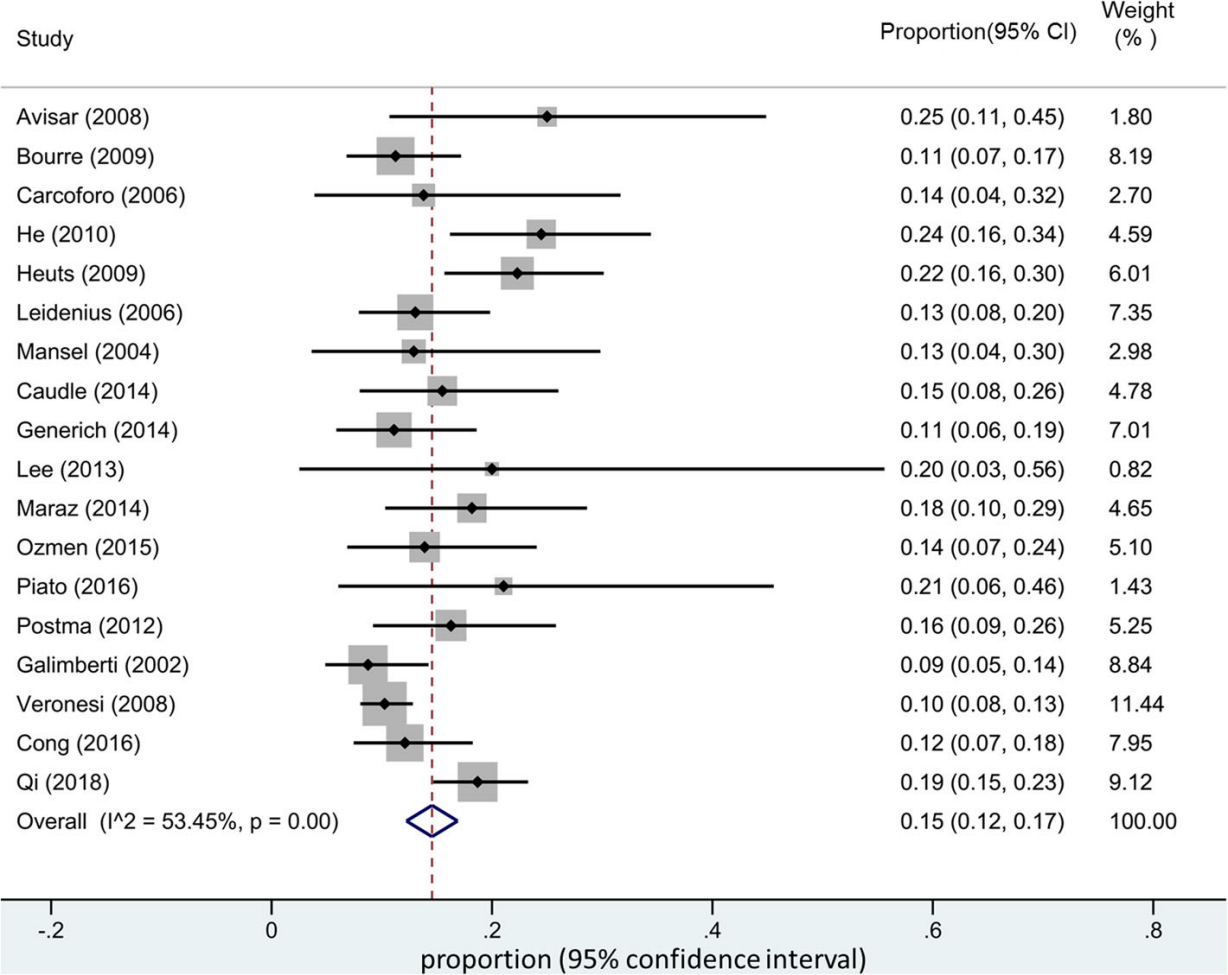
Pooled estimate of IMN-SLNB positivity rate

**Fig. 3:**
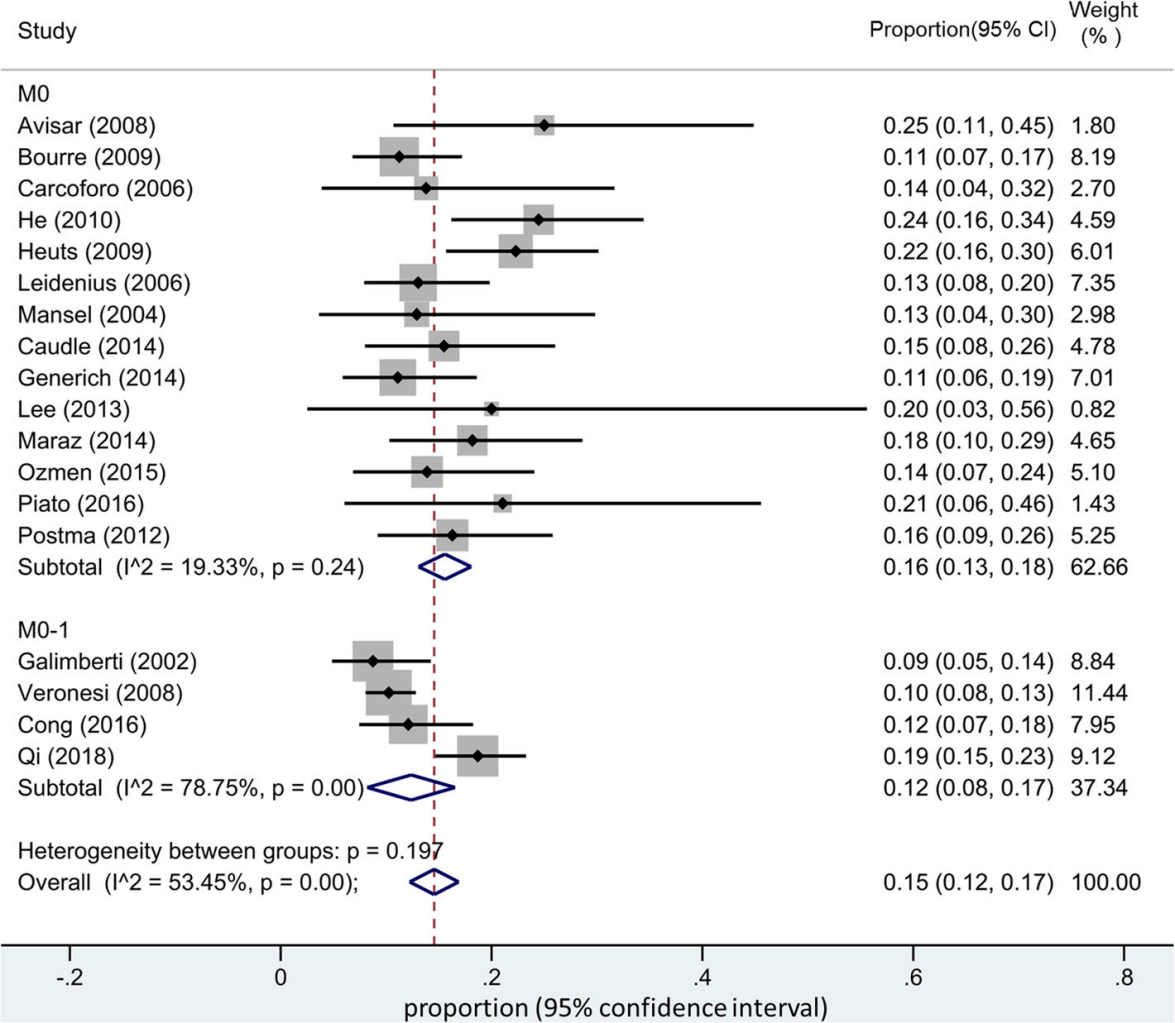
Subgroup analysis of the pooled estimate of IMN-SLNB positivity rate in patients with or without distant metastasis

**Fig. 4:**
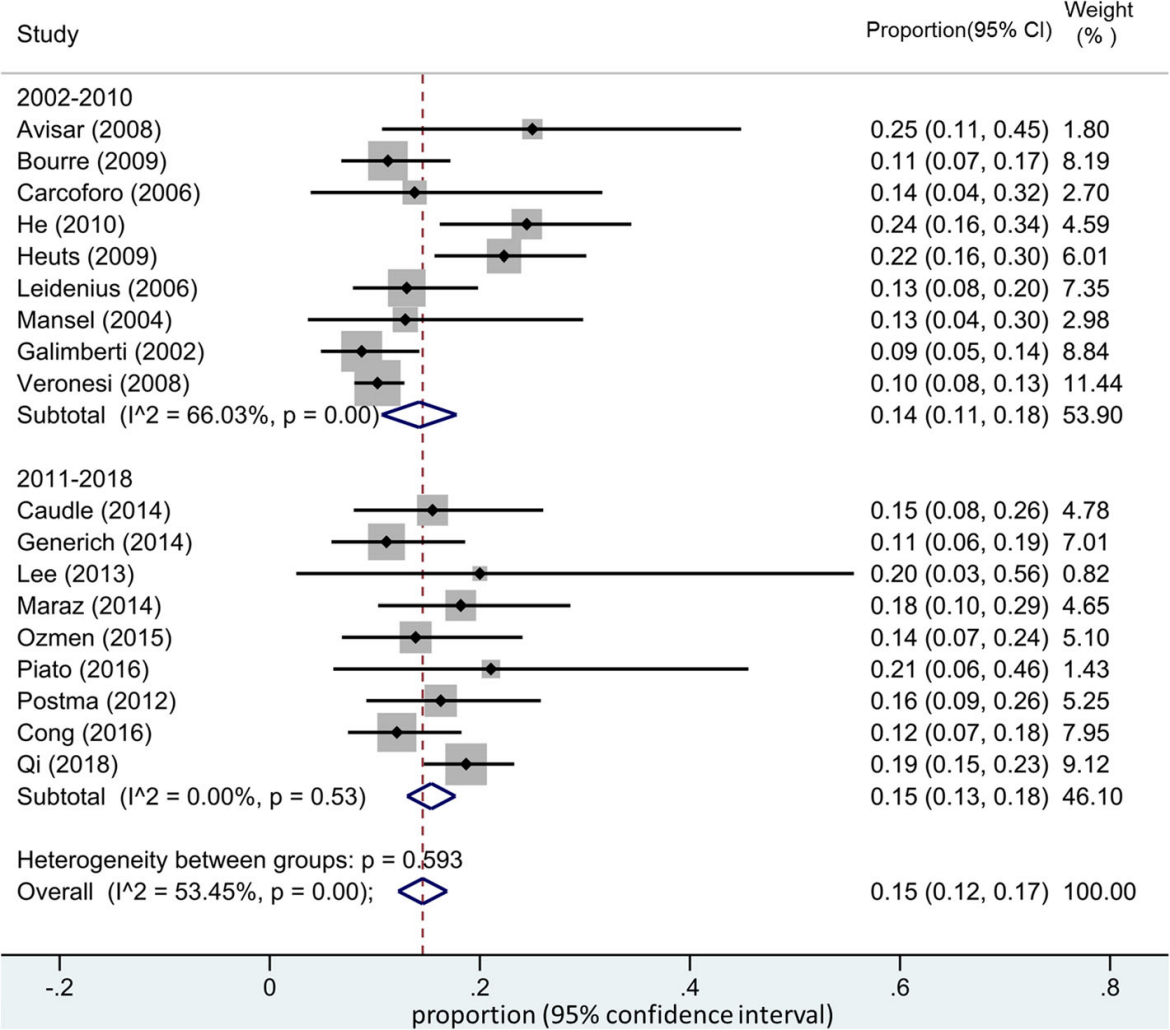
Subgroup analysis of the pooled estimate of IMN-SLNB positivity rate stratified by publication year

The pooled estimated of IMN-SLNB positivity rate was 15% (95% CI 12–17%) (Fig. 2). Significant between-group heterogeneity was observed and random-effects models were applied. Subgroup analysis was also performed. Included studies were further stratified by tumor stage (with or without metastasis) and publication year. Surprisingly, the pooled positive rate of IMN-SLNB did not change significantly according to our subgroup analysis (Figs. 3 and 4).

**Fig. 5:**
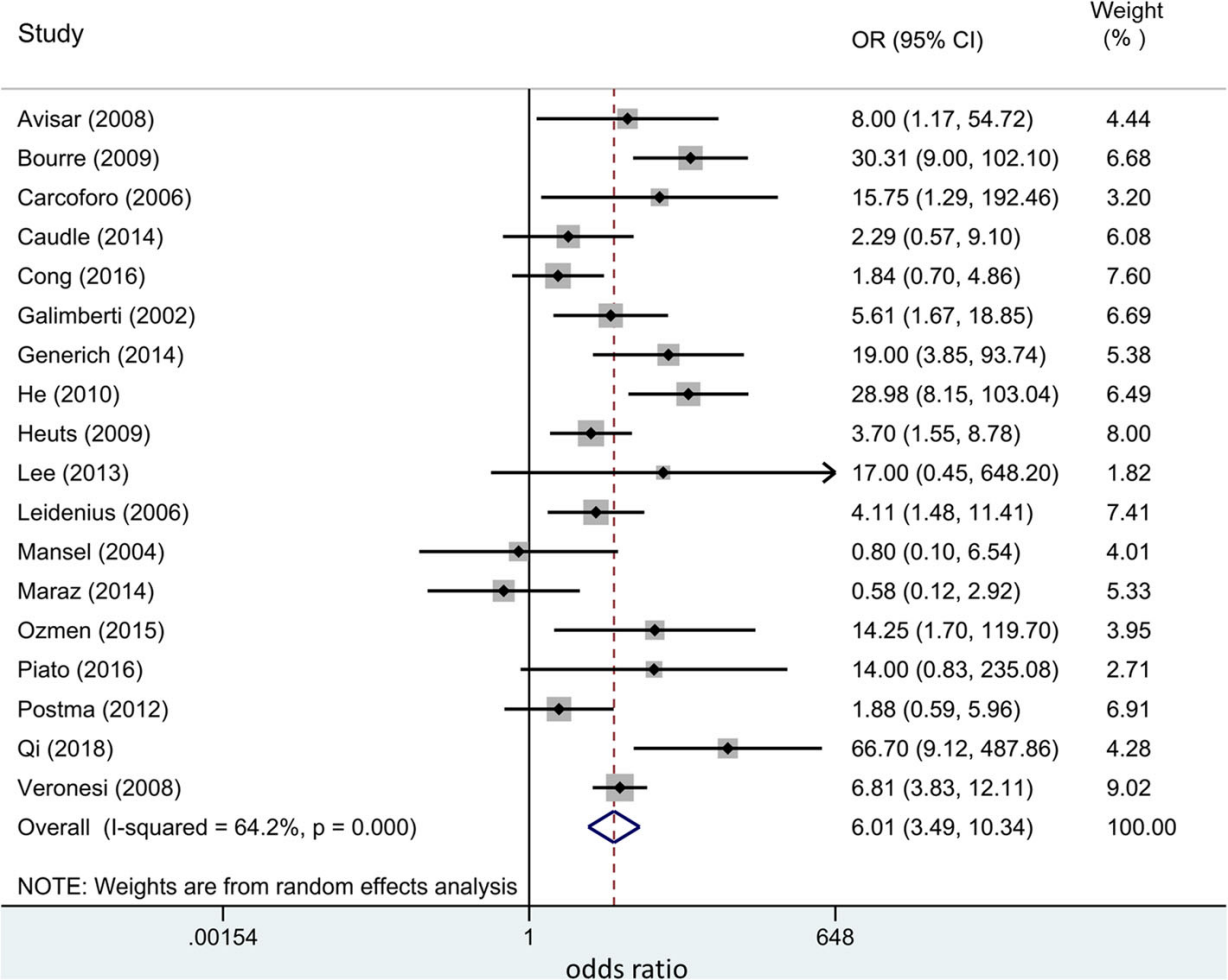
The pooled odds ratio of 4 subgroups (IMN+/AXN+, IMN+/AXN−, IMN−/AXN−, IMN−/AXN−)

We also explored the correlations of IMN and AXN metastasis. As it is shown in Fig. 5, our results indicated that axillary lymph node metastasis is a strong predictor of IMN involvement (OR 6.01, 95% CI 3.49, 10.34). The metastasis of IMN predicts the metastasis of AXN, and vice versa.

### Bias assessment

**Fig. 6:**
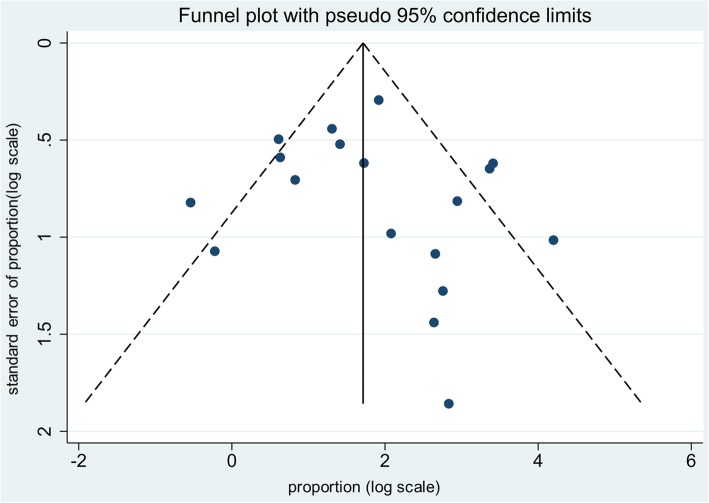
Funnel plot to assess publication bias

Funnel plot was employed to assess publication bias. As is shown in Fig. 6, there was no significant publication bias.

## Discussions

Whether we should biopsy internal mammary nodes or not has been a matter of debate for a few decades. The argument was brought up during the era of ERM when worse prognosis was noticed in patients with IMN involvement. However, the IMN dissection as part of ERM was later abandoned due to lack of survival benefit. The introduction of SLNB technique has dramatically changed the clinical management of AXN. It was also applied to evaluate IMN status since it was a relatively safe procedure compared to ERM. At first, IMN-SLNB technique was not quite reliable. Many earlier studies reported a very low incidence let alone metastasis rate of IMN. Moreover, IMN-SLNB carried a certain risk of complications. So the omission of IMN-SLNB was suggested by many researchers. Qiu et al. modified the IMN-SLNB technique and reported a visualization rate of 75% with a success rate of 98% and a serious complication rate of 9.1% [28].

The incidence of IMN metastasis might be underestimated. The incidence of IMN detected by modern imaging technique such as MRI or CT is around 45%, but it was hard to rule out reactive hyperplasia and the detection criteria varied greatly [29, 30].PET/CT is very promising in differentiating IMN SLNs from mediastinal sentinel lymph nodes (SLNs). The latter is a frequent site of tumor migration and cancer relapse, but it is unable to be biopsied without thoracic surgery [31].

Recently, several large prospective studies have demonstrated the survival benefit of RT to IMN basin [5–7]. Based on these solid evidence, the 2016 NCCN guideline has changed the level of recommendation of IMN-RT to category 2A when there is a high risk of IMN involvement. However, there are other studies that failed to show a survival benefit with IMN-RT [32, 33]. This controversy might be explained by three reasons: firstly, a pathological confirmation of IMN status was not always available in these studies. Risk stratification of IMN metastasis by clinical factors like tumor location is not always reliable. A recent study validated the hypothesis that IMN SLNs receive lymphatic drainage from all four quadrants of breast, which was consistent with results of previous studies of ERM [34]. Secondly, it was difficult to single out the survival benefit of RT to IMN basin apart from routine RT. Thirdly and most importantly, patients with IMN metastasis are still a heterogeneous sub-group of breast cancer. The 8th edition of AJCC staging system further stratified IMN-positive patients based on other prognostic factors such as hormone receptor and human epidermal growth factor receptor-2 (HER2) status, which might lead to a more accurate prognosis prediction [35]. So, it would be of great value if the status of IMN in high-risk patients could be assessed pre-operatively and adjuvant treatment plan especially radiotherapy could be tailed.

Our results showed the pooled estimated rate of metastasis IMN was 15% which was in consistence with recent reports [36]. Studies have found out that there are several factors that can affect the positivity rate of IMN-SLNB, such as tumor location, tumor stage, age, and systematic therapy. The mapping and detection procedure will also affect the visualization of IMN. The majority of our included studies employed more than more one mapping techniques. Despite the variation of visualization rate, publication year and the presence of distant metastasis, the positivity rate of IMN-SLNB was rather consistent. Our study also showed that there was a significant correlation between IMN and AXN. Patients with AXN involvement are 6 times more likely to have IMN involvement. This correlation of AXN and IMN has been noticed since the era of ERM [37]. Our results add to the growing body of evidence for the use of IM-SLNB in patients with axillary involvements [26]. However, these results should be interpreted with caution. Future work is required to verify if IMN-SLNB should be recommended in case of AXN involvement and be omitted when AXN is negative.

Our study might be subjected to several limitations. We are trying to draw a contemporary picture of IMN status in the era of SLNB. Yet, our characteristic analysis was based on data extracted from a majority of retrospective studies and therefore selective bias was inevitable. On the other hand, we conducted a rigorous screening. We were able to exclude case reports and very small case serious. However, there are some other means to investigate IMN that was not included in our study. For instance, IMN biopsy during breast reconstructions is not very uncommon, and IMN status can also be determined by fine-needle aspiration or core biopsy or thoracic surgery. Finally, only 3 of our included studies reported survivals with insufficient data; therefore, a pooled survival analysis cannot be performed and the impact of positive IM-SLNB on survival cannot be determined.

## Conclusions

Our results revealed the estimated IMN-SLNB positivity rate. The metastasis of axillary lymph nodes was a major predictive factor of IMN involvement. Patients with positive axillary lymph nodes have a higher risk of internal lymph nodes metastasis and IM-SLNB might be considered. More prospective randomized clinical trials should be conducted to fully address this issue.

## Data Availability

All data generated or analyzed during this study are included in this published article.

## Abbreviations

−: Negative, no presence of lymph node metastasis
+: Positive, presence of lymph node metastasis
AJCC: American Joint Committee on Cancer
AXN: Axillary lymph node
CI: Confidence interval
ERM: Extended radical mastectomy
IMN: Internal mammary lymph node
LS: Lymphoscintigraphy
M0: No presence of distant metastasis
*n*: Number of patients
N0: No presence of lymphatic involvement
NCCN: National Comprehensive Cancer Network
NR: Not reported
OR: Odds ratio
PET-CT: Positron emission tomography–computed tomography
RCT: Randomized clinical trial
RT: Radiation therapy
SLNB: Sentinel lymph node biopsy
Tis: Carcinoma in situ
